# Neurotoxic lesions of the anterior claustrum influence cued fear memory in rats

**DOI:** 10.3389/fpsyt.2024.1387507

**Published:** 2024-04-19

**Authors:** Tengyu Gu, Jing Dong, Jing Ge, Jialu Feng, Xiaoliu Liu, Yun Chen, Jianfeng Liu

**Affiliations:** ^1^Hubei Province Key Laboratory of Occupational Hazard Identification and Control, School of Medicine, Wuhan University of Science and Technology, Wuhan, Hubei, China; ^2^College of Life Sciences and Health, Wuhan University of Science and Technology, Wuhan, China

**Keywords:** claustrum, anxiety, fear memory, retrieval, extinction

## Abstract

**Background:**

The claustrum (CLA), a subcortical area between the insular cortex and striatum, innervates almost all cortical regions of the mammalian brain. There is growing evidence that CLA participates in many brain functions, including memory, cognition, and stress response. It is proposed that dysfunction or malfunction of the CLA might be the pathology of some brain diseases, including stress-induced depression and anxiety. However, the role of the CLA in fear memory and anxiety disorders remains largely understudied.

**Methods:**

We evaluated the influences of neurotoxic lesions of the CLA using auditory-cued fear memory and anxiety-like behaviors in rats.

**Results:**

We found that lesions of anterior CLA (aCLA) but not posterior CLA (pCLA) before fear conditioning attenuated fear retrieval, facilitated extinction, and reduced freezing levels during the extinction retention test. Post-learning lesions of aCLA but not pCLA facilitated fear extinction and attenuated freezing behavior during the extinction retention test. Lesions of aCLA or pCLA did not affect anxiety-like behaviors evaluated by the open field test and elevated plus-maze test.

**Conclusion:**

These data suggested that aCLA but not pCLA was involved in fear memory and extinction. Future studies are needed to further investigate the anatomical and functional connections of aCLA subareas that are involved in fear conditioning, which will deepen our understanding of CLA functions.

## 1 Introduction

The claustrum (CLA) is a brain region that reciprocally innervates almost all cortical areas in the mammalian brain (1). The CLA had been thought to be part of the insular cortex until later anatomical findings indicated that the CLA has unique expression patterns of molecules distinct from adjacent cortexes. The CLA is now widely accepted as a subcortical area between the insular cortex and striatum (2–5). Given the wide connection between the CLA and cortex, the CLA has been proposed to modulate cortical activity and participate in many brain functions (6, 7). However, the function of the CLA remains largely unknown.

The most popular assumption of the function of the CLA might be its involvement in consciousness, as proposed by Crick and Koch (8). They postulated that the CLA might synchronize perceptual, cognitive, and motor mortalities (8). According to this assumption, CLA lesions might lead to unconsciousness. However, due to the sheet-like topography of the CLA, it is thought to be hard to induce lesions of the CLA precisely by pharmacological or surgical methods. Notwithstanding, recent studies have implied the influences of CLA lesions on consciousness and other behaviors. For example, a study that analyzed combat veterans with penetrating traumatic brain injuries showed that claustrum damage was associated with the duration of loss of consciousness (9). A recent systematic review that analyzed the CLA lesion studies demonstrated that patients with relatively exclusive lesions of the CLA revealed diverse symptoms instead of a loss of single function (10). This study thus suggested that the CLA might participate in multiple functions or play a global role that influences multiple functions (10). However, the effects of CLA lesions on behavioral domains remain largely unknown.

In the last two decades, accumulative studies using innovative techniques of brain circuits, such as optogenetics, chemogenetics, and virus-mediated neuronal labeling, have uncovered many functions of the CLA, including sensory processing, integrations of multisensory information, cognition, and so forth (11–25). For example, it was shown that the anterior cingulate cortex (ACC) input to the CLA might be necessary for top-down control guiding action (26). In contrast, the neural projections from the CLA to ACC might participate in gating visceral pain (27). Various studies have suggested that the CLA might negatively modulate cortex activities. Electrophysiological and chemical studies indicated that the CLA inhibited the activity of the prefrontal cortex (PFC) (13, 14). Furthermore, behavioral studies showed that the CLA-PFC pathway could functionally modulate impulsive-like behaviors and reward processing (14, 28).

Given the involvement of the CLA in many brain functions, it is proposed that dysfunction or malfunction of the CLA might be the pathology of some brain diseases, such as, emotional disorders. Several studies have implied that the CLA might participate in fear and anxiety. Silencing the neural projections from the CLA to the medial entorhinal cortex during contextual fear conditioning impaired subsequent fear memory retrieval (29). Pharmacogenetic inhibition of the CLA-Prelimbic cortex circuit during contextual fear conditioning attenuated recent fear retrieval (30). Other studies also showed that stress and footshock could activate the CLA (25, 31–33). However, the role of the CLA in fear encoding and anxiety disorders remains largely understudied.

In this study, using a neurotoxic lesion method, we investigated the role of CLA in conditioned fear memory and anxiety-like behaviors in rats. We examined whether neurotoxic lesions of CLA subareas could cause anterograde or retrograde amnesia of auditory-cued fear memory. We also evaluated the influences of neurotoxic lesions of CLA subareas on behavioral performances in the open field and elevated plus-maze tasks.

## 2 Materials and methods

### 2.1 Animals

Male Sprague-Dawley rats (weighing 280-300 g at arrival) from the animal facility of Hunan Slack Jingda Experimental Animal Co., LTD were used. The rats were housed in standard polypropylene cages (2–3 animals per box), in a 12/12 h light/dark cycle (lights on at 06:30), with free access to food and water maintained at a constant room temperature of 24 °C ± 1 °C. The experiments were conducted during their dark phase. Rats were initially handled once daily for 5 consecutive days before training. Animal experiments are conducted in accordance with the relevant regulations of the Experimental Animal Ethics Committee of the School of Medicine of Wuhan University of Science and Technology (No.2024099) to reduce the number of experimental animals as much as possible.

### 2.2 Surgery

On the day of surgery, rats were anesthetized with isoflurane (5% for induction, 1-2% for maintenance, RWD Life Science Co., LTD) in oxygen alone in an anesthesia box, and mounted into a stereotaxic frame (Beijing Zhongshi Dichuang Technology Development Co., Ltd). The bregma was used as the reference point for anteroposterior and mediolateral coordinates. Stainless steel needles (outer diameter 0.4 mm, inner diameter 0.21 mm) were implanted bilaterally in the aCLA or pCLA according to the following coordinates: aCLA: angle 4°, anteroposterior (AP): -1.8 mm; mediolateral (ML): ± 4.22 mm; dorsoventral (DV): -5.92 mm; pCLA: angle 4°, AP: +1.0 mm; ML: ± 4.84 mm; DV: -6.08 mm; relative to bregma.

Lesion subjects received microinjections of 0.2 µL of NMDA (N-Methyl-D-Aspartic acid; 20 µg/µL dissolved in 0.9% physiological saline) in each stereotaxic coordinate (34, 35). The stainless-steel catheter was inserted slowly to penetrate the dura mater and its tip was positioned at the coordinates. The microinfusion was conducted at a rate of 0.1 µL/min and the micropipette was maintained for 8 additional minutes after injection to avoid NMDA spreading back up the pipette track. Sham-operated control subjects underwent the same procedures, except that they received physiological saline microinjections instead of NMDA. After the microinjections, the wound was sutured, and the animals were allowed to recover for seven days.

### 2.3 Fear conditioning

Fear conditioning was adapted from our previous studies (36–38). Fear conditioning was conducted in four experimental chambers (36 × 28 × 38cm; Zhongshidichuang Science and Technology Development Co., Ltd., Beijing, China) located in individual sound-attenuating chambers. The chambers were modified to two different contexts for behavioral tests (contexts A and B). Context A was set up as a black box with a transparent door and a fine grid floor. A background white noise (50 dB) was continuously given for context A. For Context B, a black and white striped background and a smoothed plastic floor were used. For the fear conditioning session, the rats were placed into the conditioning chambers (context A) for a standard auditory fear conditioning procedure, in which a neutral auditory tone (conditioned stimulus; CS) was paired with an aversive footshock (unconditioned stimulus; US). This conditioning session consisted of a 3-min baseline (BL) period followed by five CS (10 s, 80 dB, 2 kHz)-US (0.8 mA, 1 s) pairings with 60-s intertrial intervals (ITIs) and an additional 60-s post-shock period. Fear retrieval was conducted in context B. The retrieval session was comprised of a 3-min baseline period followed by 5×CS (10 s, 80 dB tone) presentations with 60-s ITI. After the retrieval test, rats were exposed to repeated CS [30×CS (10 s, 80 dB tone) presentations, 30 s ITI) to induce extinction. Extinction retention was then evaluated in context B one day after extinction the same as the retrieval test described above. Freezing was defined as the cessation of all bodily movements except for breathing.

### 2.4 Open field test

The open field test (OF) procedure was the same as that in our previous studies (39, 40). The test was used to assess locomotor activity and anxiety-like behavior. The test was conducted for 10 min in a dimly lit room. The apparatus consisted of a large arena measuring 72 × 72 × 36 cm (L x W x H). The rats’ behavior was recorded with a camera mounted above the arena and analyzed with Tracking Master V4.0 software (Beijing Zhongshi Dichuang Technology Development Co., Ltd). The apparatus was divided into 5×5 compartments, with the middle 3×3 areas serving as the center zone. The following parameters were analyzed: total distance traveled, distance traveled in center zone, and time spent in center zone. The apparatus was cleaned with 75% ethanol solution between rats.

### 2.5 Elevated plus-maze testing

The elevated plus-maze test (EPM) was used to assess anxiety-like behavior as described in our previous studies (39, 40). The test apparatus consisted of four black polypropylene arms (Beijing Zhongshi Dichuang Technology Development Co., Ltd). The two open arms had 0.5 cm ledges, and the two closed arms had 30 cm walls. The open arms were placed opposite of each other. The arms were 10 cm wide and 50 cm long and were placed on 65 cm tall acrylic legs. Testing occurred in a quiet, dimly lit room. At the beginning of each test, the rats were put in the center of the apparatus, facing an open arm. The rats were allowed to explore the apparatus for 5 min, and their behavior was recorded with a camera mounted above the maze. The EPM was divided into five zones (two open arms, two closed arms, and a center zone). Behavior was analyzed automatically (center-point detection) using Tracking Master V4.0 software (Beijing Zhongshi Dichuang Technology Development Co., Ltd). The following behavioral parameters were automatically determined: duration and entries into the open arms, closed arms, and total distance traveled. The percentage of open arm entries (open arm entries/total arm entries) and percentage of time on the open arms (time on open arms/total time on the arms) were calculated. The apparatus was cleaned with 75% ethanol solution between rats.

### 2.6 Histology

Upon completion of the experiment, rats were deeply anesthetized with 4% Chloral hydrate. (Aladdin,40 mg/mL, i.p.) and perfused transcardially with physiological saline followed by 4% formalin. Brains were extracted and stored about 16-18 h (at 4° C) in 4% formalin, after which they were transferred to a 30% sucrose solution for a minimum of 5 days. Brains were then sectioned using a cryostat (Leica CM 1950) at -20° C. The brain sections (30-µm thick) were stained with Nissl staining. The histological slides were analyzed by an experimenter blind to the animals’ behavioral performance.

### 2.7 Data analysis

All results were presented as mean ± SEM and analyzed by the GraphPad Prism 10 software (GraphPad Software, San Diego, CA). Behavioral data was analyzed using two-way repeated-measures ANOVA and unpaired *t*-tests. *Post hoc* Bonferroni’s multiple comparisons test was conducted only if F in ANOVA achieved the necessary level of statistical significance (*p* < 0.05) and there was no significant variance inhomogeneity (which precludes the use of parametric statistics). *p* < 0.05 was considered statistically significant.

## 3 Results

### 3.1 Neurotoxic lesion of the anterior but not posterior CLA causes anterograde amnesia

**Figure 1:**
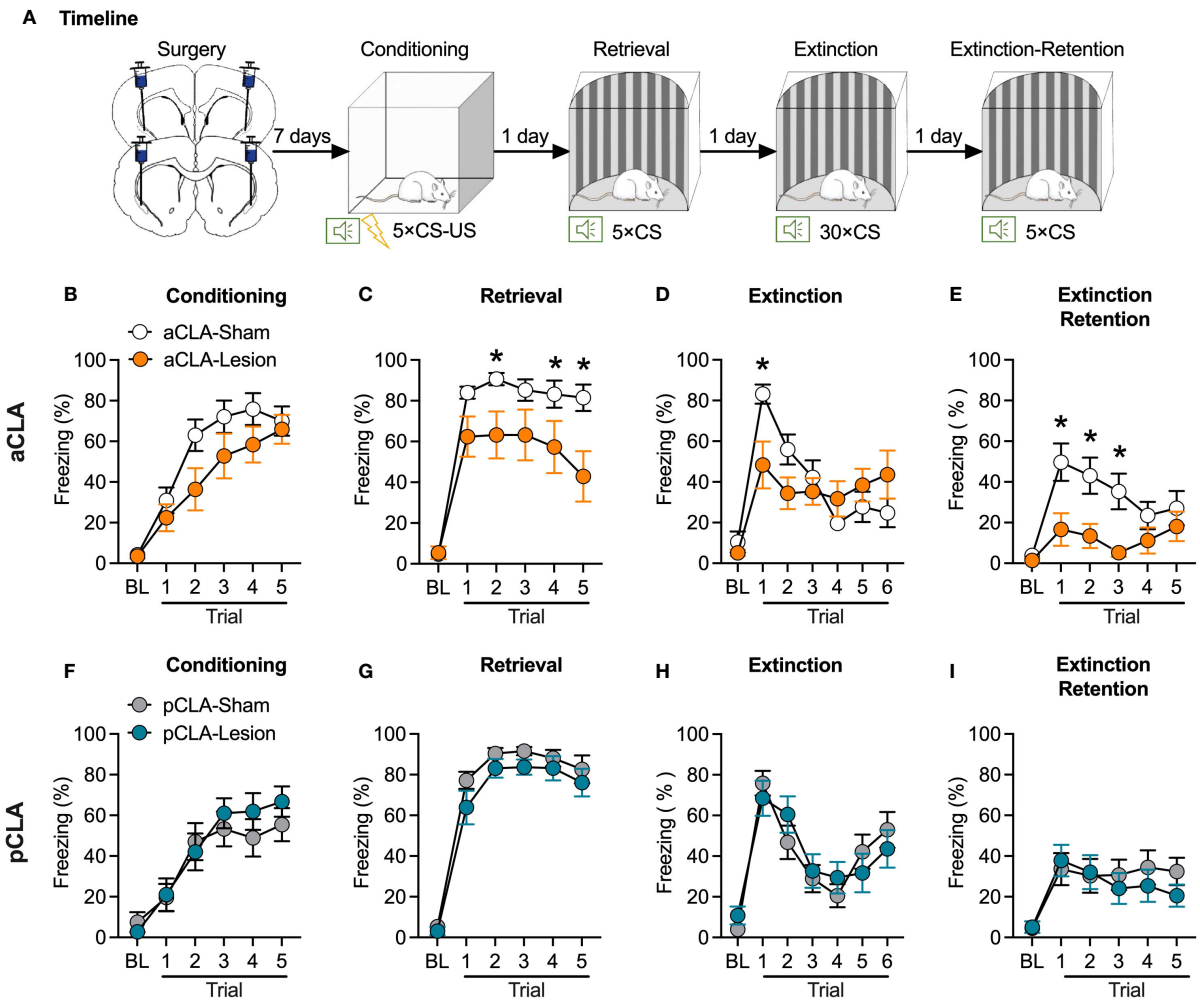
Neurotoxic lesion of the anterior but not posterior CLA causes anterograde amnesia. **(A)** Timeline for the experiment. **(B–E)** Pre-learning aCLA lesions did not affect freezing behavior during fear conditioning **(B)**, and extinction **(E)**, but reduced freezing level during fear retrieval **(C)** and extinction retention test **(E)**. **(F–I)** Pre-learning pCLA lesions did not affect freezing behavior during fear conditioning **(F)**, retrieval **(G)**, extinction **(H)**, and extinction retention test **(I)**. aCLA, anterior claustrum; pCLA, posterior claustrum. Data are expressed as mean ± SEM; n = 10-13/group; *p < 0.05.

In experiment 1, to determine the role of the claustrum in fear learning and memory, we evaluated the influences on the acquisition, retrieval, and extinction of auditory fear memory after the CLA lesion. As illustrated in **Figure 1A**, rats received a microinjection of the glutamatergic agonist NMDA (0.2 µL/side) into the anterior (aCLA) or posterior (pCLA) claustrum to induce pharmacological lesion during the surgery (aCLA-Lesion and pCLA-Lesion groups). Saline (0.2 µL/side) was microinjected into the aCLA or pCLA for control groups (aCLA-Sham and pCLA-Sham groups). Rats with aCLA or pCLA lesions showed normal activities in the home cage and were healthy overall. After recovery from the surgery (7 days), all rats underwent auditory fear conditioning (5 × CS-US associations; CS: 10 s, 80 dB, 2 kHz tone; US: 0.8 mA, 1 s footshock) in context A. Twenty-four hours later, rats were placed into Context B and received five presentations of CS tones after 3-min baseline period. Conditioned fear was then extinguished in context B, and cued fear memory was re-evaluated 24 hours after extinction as the extinction retention test (**Figure 1A**). Freezing behavior was used as the index to assess fear memory. Two-way repeated-measures ANOVA was conducted for fear conditioning, retrieval, extinction, and extinction retention.

For the pre-learning aCLA lesion experiment, ANOVA for fear conditioning revealed a significant main effect of trial (*F*_5, 105_ = 55.37, *p* < 0.05) but no significant effect of lesion (*F*_1, 21_ = 2.28, *p* > 0.05) or interaction of lesion × trial (*F*_5, 105_ = 2.04, *p* > 0.05; **Figure 1B**), suggesting that all groups of rats showed similar learning rate of auditory fear. This data indicates that CLA lesion rats showed normal learning ability. For fear retrieval, there were significant main effects of trial (*F*_5, 105_ = 86.49, *p* < 0.05) and lesion (*F*_1, 21_ = 5.62, *p* < 0.05), and a significant interaction of lesion × trial (*F*_5, 105_ = 4.74, *p* < 0.05; **Figure 1C**). As shown in **Figure 1C**, the aCLA-Lesion group showed a lower freezing level than the aCLA-Sham group during all trials of cue presentations. Rats were then repeatedly exposed to CS in context B to extinguish learned fear. For extinction, two-way ANOVA analysis revealed no main effect of lesion (*F*_1, 21_ = 0.59, *p* > 0.05) but a significant effect of trial (*F*_6, 126_ = 12.65, *p* < 0.05) and lesion × trial interaction (*F*_6, 126_ = 3.78, *p* < 0.05), indicating that the two groups of rats achieved similar levels of extinction (**Figure 1D**). For the extinction retention test, ANOVA showed significant main effects of lesion (*F*_1, 21_ = 11.46, *p* < 0.05) and trial (*F*_5, 105_ = 5.26, *p* < 0.05) but no interaction of lesion × trial (*F*_5, 105_ = 2.03, *p* > 0.05). *Post hoc* analysis revealed that compared to the aCLA-Sham group, the aCLA-Lesion group showed lower freezing levels during the first three trials of the extinction retention test (all *p* < 0.05; **Figure 1E**).

In contrast, pre-learning lesion of the pCLA did not affect fear conditioning (main effect of lesion, *F*_1, 25_ = 0.24, *p* > 0.05; interaction of lesion × trial, *F*_5, 125_ = 0.97, *p* > 0.05; **Figure 1F**), or fear retrieval (main effect of lesion, *F*_1, 25_ = 0.28, *p* > 0.05; interaction of lesion × trial, *F*_5, 125_ = 0.37, *p* > 0.05; **Figure 1G**). In addition, compared to the pCLA-Sham group, the pCLA-Lesion showed similar freezing level during the extinction (main effect of lesion, *F*_1, 25_ = 0.02, *p* > 0.05; interaction of lesion × trial, *F*_6, 150_ = 1.04, *p* > 0.05; **Figure 1H**) and extinction retention test (main effect of lesion, *F*_1, 25_ = 0.31, *p* > 0.05; interaction of lesion × trial, *F*_5, 125_ = 0.60, *p* > 0.05; **Figure 1I**). These data indicate that neurotoxic lesions of the aCLA but not pCLA disrupt auditory-cued fear retrieval and promote fear extinction.

### 3.2 Post-learning neurotoxic lesion of the anterior but not posterior CLA facilitates fear extinction

**Figure 2:**
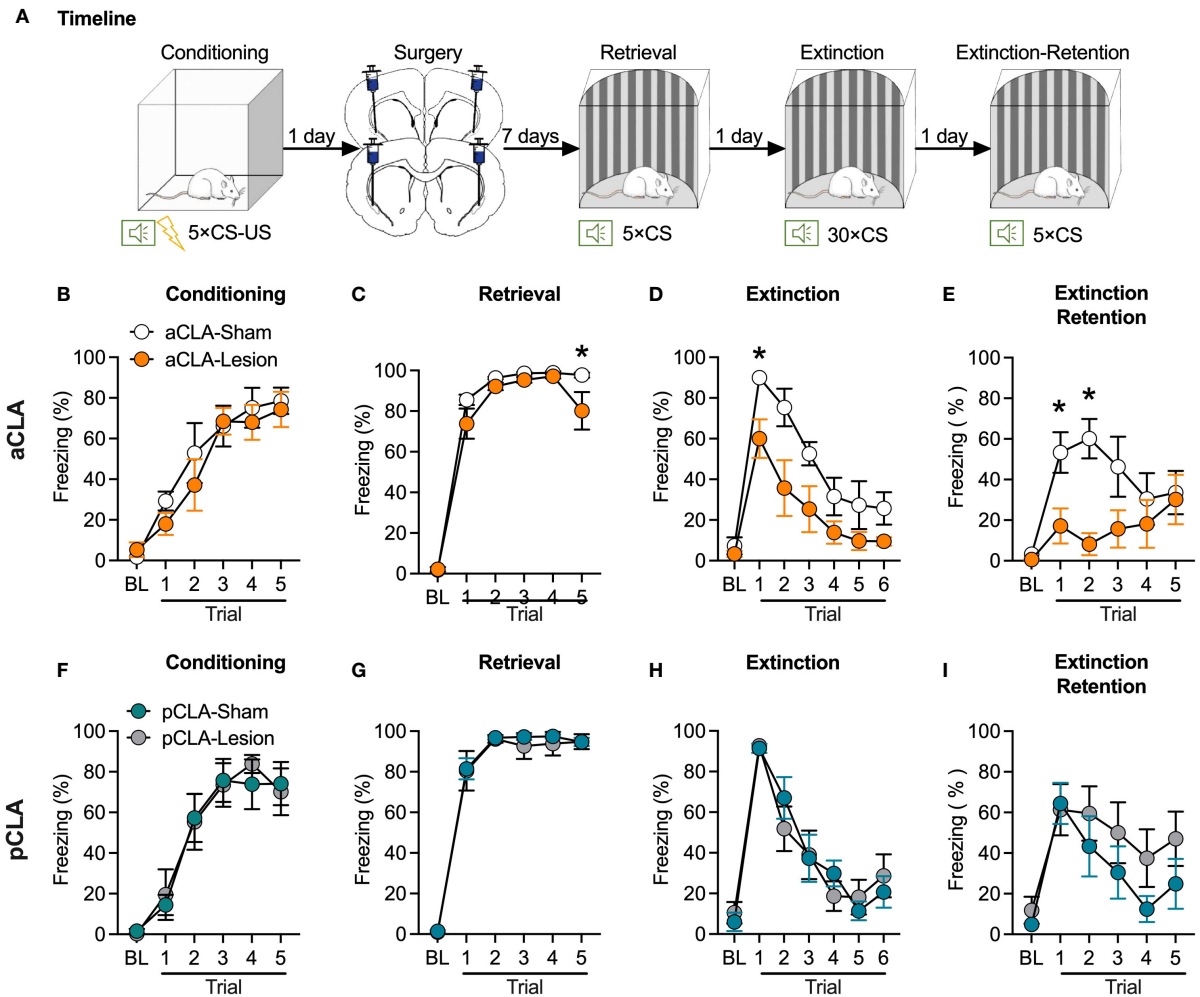
Post-learning neurotoxic lesion of the anterior but not posterior CLA facilitates fear extinction. **(A)** Timeline for the experiment. **(B)** Rats that were assigned to the aCLA-Sham and aCLA-Lesion groups showed similar levels of fear learning. **(C–E)** Post-learning aCLA lesions reduced freezing level in the last but not other retrieval trials **(C)**, facilitated extinction **(D)**, and decreased freezing levels in the extinction retention test **(E)**. **(F–I)** Post-learning pCLA lesions did not affect freezing behavior during fear conditioning **(F)**, retrieval **(G)**, extinction **(H)**, and extinction retention test **(I)**. aCLA, anterior claustrum; pCLA, posterior claustrum. Data are expressed as mean ± SEM; n = 6-7/group; *p < 0.05.

The findings above suggest that CLA lesions could cause anterograde amnesia of fear memory. In experiment 2, we evaluated fear retrieval and extinction after a post-conditioning lesion of the claustrum to examine whether CLA lesions might induce retrograde amnesia. The surgery and behavioral procedures were similar to those described above, except that neurotoxic lesions were conducted after rather than before fear conditioning (**Figure 2A**). Rats underwent surgery one day after fear conditioning and were allowed for subsequent one-week recovery. Therefore, fear retrieval test was conducted eight days after fear conditioning.

For post-learning aCLA lesion, there was no difference between the two groups during fear conditioning (main effect of lesion, *F*_1, 11_ = 0.44, *p* > 0.05; interaction of lesion × trial, *F*_5, 55_ = 0.58, *p* > 0.05; **Figure 2B**). For the retrieval, ANOVA analysis showed significant main effects of lesion (*F*_1, 11_ = 17.78, *p* < 0.05) and trial (*F*_5, 55_ = 225.5, *p* < 0.05) but not significant lesion × trial interaction (*F*_5, 55_ = 1.92, *p* > 0.05; **Figure 2C**). Interestingly, *post hoc* analysis indicated that lesion rats showed relatively lower freezing during the last retrieval trial but not in other retrieval trials. These results suggested that the lesion rats might show regular retrieval but faster extinction than the sham rats. Consistently, statistical analysis conducted for extinction showed significant main effects of lesion (*F*_1, 11_ = 9.85, *p* < 0.05) and trial (*F*_6, 66_ = 25.92, *p* < 0.05) but not significant lesion × trial interaction (*F*_6, 66_ = 1.43, *p* > 0.05; **Figure 2D**). *Post hoc* analysis indicated that compared to the aCLA-Sham group, the aCLA-Lesion group showed lower levels of freezing in the first (*t*_11_ = 2.65, *p* = 0.07) and second (*t*_11_ = 3.50, *p* < 0.05) trial of extinction. The aCLA-Lesion group also showed lower levels of freezing during the extinction retention test [main effects of lesion (*F*_1, 11_ = 5.13, *p* < 0.05) and trial (*F*_5, 55_ = 5.21, *p* < 0.05) and a significant interaction of lesion × trial (*F*_5, 55_ = 3.27, *p* < 0.05)]. *Post hoc* analysis revealed lower levels of freezing of aCLA-Lesion rats than the aCLA-Sham rats in the first two trials of the extinction retention test (first trial, *t*_11_ = 2.54, *p* = 0.08; second trial, *t*_11_ = 3.65, *p* < 0.05; **Figure 2E**).

For post-learning pCLA lesion, the two groups of rats acquired similar levels of fear during conditioning (main effect of lesion, *F*_1, 11_ = 0.01, *p* > 0.05; interaction of lesion × trial, *F*_5, 55_ = 0.23, *p* > 0.05; **Figure 2F**). Similar to the results of pre-learning lesion of pCLA, post-learning lesion of the pCLA did not affect fear retrieval (main effect of lesion, *F*_1, 11_ = 0.14, *p* > 0.05; interaction of lesion × trial, *F*_5, 55_ = 0.21, *p* > 0.05; **Figure 2G**), extinction (main effect of lesion, *F*_1, 11_ = 0.01, *p* > 0.05; interaction of lesion × trial, *F*_6, 66_ = 0.73, *p* > 0.05; **Figure 2H**), or extinction retention test (main effect of lesion, *F*_1, 11_ = 2.17, *p* > 0.05; interaction of lesion × trial, *F*_5, 55_ = 0.53, *p* > 0.05; **Figure 2I**). Taken together, these data indicate that post-conditioning neurotoxic lesions of the aCLA but not pCLA facilitated fear extinction.

### 3.3 Neurotoxic lesion of the anterior or posterior CLA did not affect locomotion or anxiety-like behaviors

**Figure 3:**
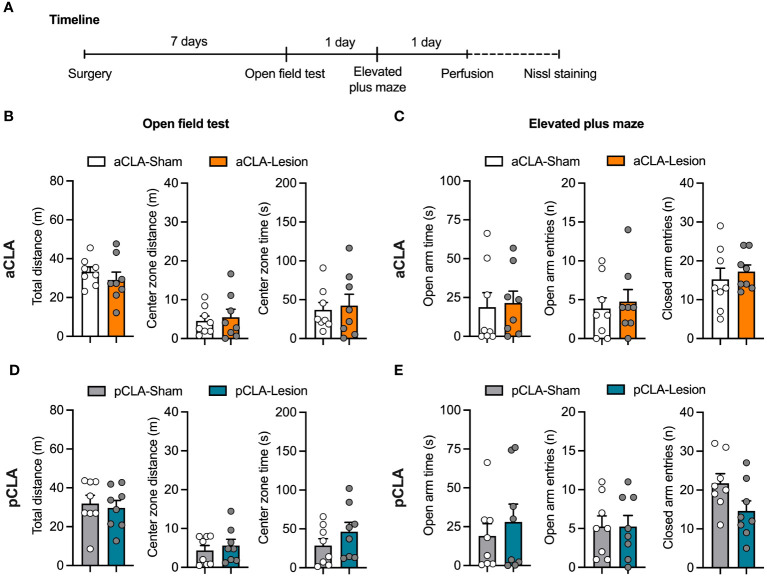
Neurotoxic lesion of the anterior or posterior CLA did not affect locomotion or anxiety-like behaviors. **(A)** Timeline for the experiment. **(B, C)** aCLA and pCLA lesions did not affect behavioral performances in the open field test. **(D, E)** aCLA and pCLA lesions did not affect behavioral performances in the elevated-plus maze test. aCLA, anterior claustrum; pCLA, posterior claustrum. Data are expressed as mean ± SEM; n = 8/group; *p < 0.05.

To examine whether chemical lesions of the claustrum could affect general anxiety, we evaluated anxiety-like behaviors after aCLA and pCLA lesions by the open field and elevated plus-maze tests. Seven days after NMDA or saline microinjection into the aCLA (aCLA-Lesion and aCLA-Sham groups) or pCLA (pCLA-Lesion and pCLA-Sham groups), rats underwent the behavioral tests (**Figure 3A**). Unpaired *t*-test analysis was conducted for each parameter in the two behavioral tests. For the open field test, no difference was found for total distance traveled (aCLA: *t*_14_ = 0.89, *p* > 0.05; pCLA: *t*_14_ = 0.38, *p* > 0.05), distance traveled in the center zone (aCLA: *t*_14_ = 0.40, *p* > 0.05; pCLA: *t*_14_ = 0.63, *p* > 0.05), or time spent in the center zone (aCLA: *t*_14_ = 0.32, *p* > 0.05; pCLA: *t*_14_ = 1.19, *p* > 0.05; **Figures 3B, C**). For the elevated plus-maze, there was no statistical difference between two groups for time spent in open arms (aCLA: *t*_14_ = 0.22, *p* > 0.05; pCLA: *t*_14_ = 0.65, *p* > 0.05), entries into open arms (aCLA: *t*_14_ = 0.42, *p* > 0.05; pCLA: *t*_14_ < 0.01, *p* > 0.05), or entries into closed arms (aCLA: *t*_14_ = 0.60, *p* > 0.05; pCLA: *t*_14_ = 2.00, *p* > 0.05; **Figures 3D, E**). These data suggested that lesions of aCLA or pCLA did not affect general locomotion or anxiety-like behaviors.

**Figure 4:**
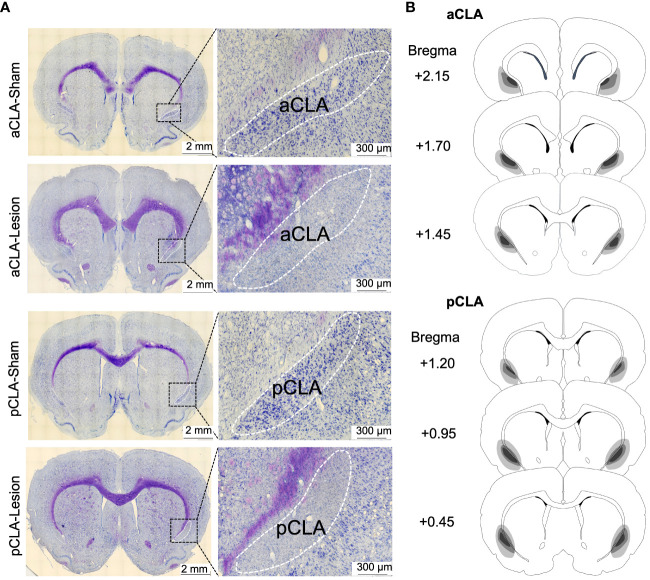
Examples and schematic representations of CLA sections with lesions. **(A)** Example images of brain sections with CLA lesions. **(B)** Schematic representations of lesion areas in the CLA in the study.

After behavioral experiments, brain sections were stained with Nissl staining after behavioral testing in all experiments. As depicted in **Figure 1**, NMDA caused extensive loss of neurons in the anterior or posterior CLA (aCLA or pCLA; **Figure 4**).

## 4 Discussion

The current study demonstrated that pharmacological lesions of the aCLA before fear conditioning suppressed freezing response during cued fear retrieval and extinction, indicating an anterograde amnesia. In contrast, pharmacological lesions of the aCLA after fear conditioning did not affect cued fear retrieval but facilitated fear extinction. Pharmacological lesions of the pCLA neither before nor after fear conditioning affected cued fear memory. Lesions of the aCLA or pCLA did not affect anxiety-like behaviors evaluated by the open field and elevated-plus maze tests. Together, the current study suggested that aCLA but not pCLA played an essential role in auditory-cued fear memory.

Crick and Koch proposed that the CLA might be crucial for consciousness. Notwithstanding recent evidence that rodents could pass the mirror test to determine the ability to be self-aware, it remains debated whether rodents are mentally conscious. Nevertheless, some animal studies have implied a contribution of the CLA in sleep/wakefulness transition and anesthesia, behavioral states that might be related to consciousness (15, 16, 41–43). For example, optogenetic activation of the CLA could induce general long-lasting silencing of the frontal cortex during slow-wave sleep and quiet wakefulness (15). In contrast, genetic ablation of the CLA could attenuate slow-wave activity (15). Findings on the role of CLA in anesthesia are somehow controversial. A study showed that electrical stimulation of the CLA could deepen isoflurane anesthesia (42). However, another study reported that chemogenetic activation of the CLA induced attenuated propofol sensitivity and shorter anesthesia duration, which might be mainly mediated by local GABAA receptors (43). In the present study, we found that rats with lesions of aCLA or pCLA showed normal movement activities in the OF and EPM tasks and no sign of unhealthy conditions, indicating that they were in at least mental consciousness. Nevertheless, we did not examine other brain functions that may be more directly linked to mental consciousness, such as sleep/wakefulness transactions. Alternatively, consciousness may need fine coordination of functional modalities rather than being produced by a single core brain area. As proposed in a previous study, CLA lesions could influence many brain functions, including cognition, perception, mental state, and sleep.

A few studies have implicated the role of CLA in fear memory. Kitanishi and Matso showed that optogenetic silencing of the medial entorhinal cortex (MEC)-projecting CLA neurons during contextual fear conditioning attenuated later fear retrieval (29). Consistent with these, a recent study showed that footshock could activate the MEC-projecting CLA neurons (31). Interestingly, these studies also showed that novel context but not a neural auditory cue could activate the MEC-projecting CLA neurons (29, 31). Another study showed that chemogenetic silencing the neural projections from the CLA to the prelimbic cortex (PL) during contextual fear conditioning attenuated subsequent retrieval of recent but not remote fear memory (30). In the present study, pre-learning lesions of CLA did not affect the acquisition of auditory-cued fear conditioning but attenuated subsequent retrieval. These data suggest that particular brain area-projecting CLA neurons could be activated by footshock during conditioning to be engaged in fear memory learning/encoding, which eventually determines the subsequent retrieval. This was further supported by our data that post-learning lesion of anterior CLA did not affect fear retrieval. Another consideration could be that the CLA might be involved in the recent but not remote fear. A previous study showed that chemogenetic inhibition of PL-projecting CLA neurons only attenuated recent but not remote contextual fear retrieval (30). In the post-learning lesion experiment (**Figure 2**) in this study, fear retrieval was tested nearly a week after fear conditioning to ensure recovery from surgery. Therefore, it is unknown whether post-learning lesions of the CLA might affect a more recent memory retrieval, which requires further investigation. The claustrum has also been implicated in fear extinction. The CLA has abundant neural projections to the infralimbic cortex (IL), a brain region that plays a crucial role in fear extinction. A study showed that the activity of CLA neurons was elevated after successful fear extinction (44). However, retrieval of extinction memory did not activate the IL-projecting CLA neurons, suggesting that the CLA might participate in fear extinction via modulating brain areas other than the IL. The present study showed that post-learning lesions of the aCLA selectively facilitated fear extinction without influencing fear retrieval. It was proposed that extinction is a new learning process that shares many mechanisms of fear learning. We speculate that the aCLA lesion might affect extinction learning and retrieval.

The CLA has been linked to stress-related disorders, including anxiety and depression. Recent studies showed that acute restraint stress and social defeat stress could activate claustral neurons and neurons project to the CLA and basolateral amygdala (32, 33). Chemogenetic activation and inhibition of the stress-responsive CLA neurons could induce and ameliorate anxiety-like behaviors, respectively (32). Furthermore, optogenetic activation of stress-responsive BLA terminals in the CLA could induce anxiety-like behaviors (32). Chemogenetic inhibition of the stress-responsive CLA neurons could attenuate chronic social defeat stress-induced depressive-like behaviors (32). These findings thus suggest that stress activates the CLA, and the CLA positively mediates stress response. However, findings from these studies are partly controversial, with a recent report which showed that the CLA might negatively modulate stress response (24). The study demonstrated that chemogenetic inhibition and activation of glutamatergic neurons in the CLA facilitated and attenuated social defeat stress-induced depressive-like behaviors (25). It was further revealed that the CLA glutamatergic neurons might regulate depressive-like behaviors via targeting parvalbumin-positive interneurons in the PL (25). These findings are consistent with previous studies that demonstrated that the CLA negatively controls the prefrontal cortex (13, 14). It is possible that the discrepancies might be caused by interventions on different types of claustral neurons in these studies. Nevertheless, these studies provided the pilot evidence that the CLA might be involved in stress response and stress-induced anxiety- and depressive-like behaviors. In contrast, our present data showed that lesions of the aCLA or pCLA had no effect on anxiety-like behaviors. It should be noted that, unlike most of the above-mentioned studies, we only evaluated the influences in non-stressed animals. Future studies are needed to examine whether CLA lesions might affect anxiety-like behaviors under stressful conditions.

Interestingly, our data showed that aCLA but not pCLA is crucial for fear memory, suggesting subarea-specific functional involvement of the CLA in fear memory. This is consistent with the evidence that different proportions of the CLA might be differentially involved in certain behaviors (45, 46). For example, a recent study showed that medial CLA was important for anxiety-like behaviors, whereas the aCLA participated in cocaine reward memory in adult animals that were exposed to cocaine during adolescence (46). The pCLA but not aCLA was involved in exercise intensity-dependent cardiovascular regulation (45). The differential roles of anterior and posterior parts of the CLA could be due to their different anatomical or functional connections with cortical and subcortical areas. A study showed that the frontal cortices and sensorimotor cortices primarily target the ACC-projecting neurons in the anterior but not posterior CLA (47).

In summary, the present study indicated a critical role of aCLA in auditory-cued fear memory and extinction. Future studies are needed to further investigate the anatomical and functional connections of CLA subareas that are differentially involved in fear memory storage and cognition, which will deepen our understanding of CLA functions.

## Data availability statement

The raw data supporting the conclusions of this article will be made available by the authors, without undue reservation.

## Ethics statement

The animal study was approved by Experimental Animal Ethics Committee of the School of Medicine of Wuhan University of Science and Technology. The study was conducted in accordance with the local legislation and institutional requirements.

## Author contributions

TG: Writing – review & editing. JD: Writing – review & editing. JG: Writing – review & editing. JF: Writing – review & editing. XL: Writing – review & editing. YC: Writing – review & editing. JL: Conceptualization, Data curation, Formal Analysis, Funding acquisition, Project administration, Resources, Supervision, Writing – original draft.
